# Prenatal Exposure to Alcohol: What the Images Reveal

**Published:** 1995

**Authors:** Sarah N. Mattson, Edward P. Riley

**Affiliations:** Sarah N. Mattson, Ph.D., is associate director and Edward P. Riley, Ph.D., is director of the Center for Behavioral Teratology, San Diego State University, San Diego, California

**Keywords:** fetal alcohol syndrome, prenatal alcohol exposure, gestation, magnetic resonance imaging, brain damage, child, fetal alcohol effects

## Abstract

Case studies and statistical analyses of groups of children with fetal alcohol syndrome (FAS) or prenatal exposure to alcohol (PEA) have been performed using magnetic resonance imaging (MRI) techniques. These studies show that alcohol exposure during pregnancy can result in a range of structural brain abnormalities. Among severely affected children, widespread damage may occur to the corpus callosum, basal ganglia, hippocampus, and cerebellum. Throughout the range of severity, however, overall reduction of the brain is apparent, and in particular, portions of the cerebellum, basal ganglia, and corpus callosum are disproportionately reduced in size among children with FAS and PEA. These children also have been shown to have cognitive deficits, although these have not been linked to abnormalities in specific brain areas.

The effects of exposure to alcohol during the prenatal period have been studied intensively using a variety of research methods, including autopsies, animal models, and imaging techniques. Through these efforts, investigators have learned that prenatal alcohol exposure^1^ can have devastating effects on the developing fetus and that these effects can be viewed as falling along a continuum. At the farthest end of the continuum are perinatal death and fetal alcohol syndrome (FAS) (see Jones and Smith 1973). An FAS diagnosis is made when an alcohol-exposed infant exhibits pre- or postnatal growth deficiency, a specific pattern of anomalies in the structure of the face and skull (i.e., craniofacial anomalies), and some central nervous system dysfunction (see figure 1). In addition to causing FAS, prenatal alcohol exposure can result in a variety of other severe effects, such as mental retardation and additional physical anomalies. But lesser cognitive deficits and behavioral difficulties related to prenatal alcohol exposure can be apparent even at the mild end of the continuum (see Jacobson and Jacobson 1994). For example, researchers have reported that exposure to even small amounts of alcohol (1–2 drinks per day) during prenatal development can lead to impaired attention (for review, see Streissguth 1986). The terms “fetal alcohol effects” (FAE) and “prenatal exposure to alcohol” (PEA) are sometimes used to describe people who do not exhibit all the features required for a diagnosis of FAS but for whom some physical or behavioral problems are ascribed to prenatal alcohol exposure.

Although behavioral and developmental anomalies have been documented in children prenatally exposed to alcohol, no one has yet defined precisely what brain structures specifically are affected following such exposure. Researchers are beginning to apply imaging techniques—such as magnetic resonance imaging (MRI), which produces pictures of the brain’s structure—to investigate this question. MRI studies have shown that structures such as the cerebral cortex, cerebellum, basal ganglia, hippocampus, and corpus callosum appear to be affected by PEA. The cerebral cortex is the outer part of the brain and is necessary for a variety of higher cognitive functions; the cerebellum is found at the back of the brain and is involved in motor as well as cognitive activities; the basal ganglia are a group of structures that lie deep within the brain (under the cortex) and are involved in motor and cognitive skills. The hippocampus is involved in memory, and the corpus callosum is the tract of nerve fibers bridging the two brain hemispheres.^2^ This article reviews MRI findings that reveal a pattern of structural anomalies in the brains of children with FAS or PEA. Such research eventually may be linked to specific developmental and behavioral deficits observed in these children to clarify the nature of this disorder and discern the true capabilities of those who suffer from it.

## Documenting Severe Brain Damage

### Autopsy Reports of Children With FAS

The effects of prenatal alcohol exposure on the developing brain have received a great deal of attention since alcohol was first implicated in causing damage to the unborn fetus (i.e., it is a teratogen) more than 20 years ago. In 1973, when the first cases of FAS were documented, an autopsy was reported detailing severe and widespread brain abnormalities. These anomalies included failure of the young nerve cells (i.e., neurons) to migrate during development to their appropriate locations in the brain, absence (i.e., agenesis) of the corpus callosum, and general disorganization of the brain tissue. Since that initial case, additional autopsy reports on severely affected children have documented a variety of brain abnormalities (Clarren 1986). Among these is the abnormal development of specific brain areas, such as the cerebellum, corpus callosum, and basal ganglia. Other anomalies associated with alcohol include an increase of fluid in the brain (i.e., hydrocephaly), abnormally small brain size (i.e., microcephaly), and a complete absence of higher brain structures (i.e., anencephaly). (A review of the brain damage associated with prenatal alcohol exposure can be found in Mattson and Riley in press).

### Animal Studies

In addition to autopsy reports, other information about alcohol’s effects on the developing brain is available from research using animal models. For example, in mice, prenatal alcohol exposure has been associated with a developmental disorder called holoprosencephaly, which has a range, or spectrum, of severities. In the most severe form of holoprosencephaly, the two hemispheres of the brain fail to separate, signifying a severe defect in brain development. In milder cases, at least a partial separation of the two hemispheres occurs, although midline structures, such as the corpus callosum, may still be abnormal. In rats, abnormal development of the hippocampus, cerebellum, and basal ganglia also have been noted following perinatal alcohol exposure. (For a review of the effects of alcohol on brain development in animals, see Miller 1992).

## Magnetic Resonance Imaging of Children With FAS

One difficulty in using autopsy reports to generalize the type and extent of brain damage resulting from prenatal alcohol exposure is that most of these children died because of the extent of their alcohol-related physical problems and, therefore, are unlikely to convey an accurate picture of alcohol’s teratogenicity across the continuum. Instead, the autopsy cases likely demonstrate the most extreme effects that alcohol can have on the developing brain—that is, profound disorganization that may result in death. Although the information obtained from these autopsies remains extremely valuable in the study of FAS, a few recent reports that make use of brain imaging techniques to evaluate children who more closely represent most of the FAS and FAE or PEA cases appear to take knowledge of the full range of alcohol-related brain anomalies a step further.

Over the past 4 years, MRI has been used with some success in FAS and PEA children. MRI is a noninvasive procedure in which a powerful magnet is used to align the atoms in the brain. These atoms absorb energy when an electromagnetic signal is passed through the magnetic field. When the electromagnetic signal is turned off, the atoms “relax” and release energy. The measurement of this energy is used to create pictures of the brain. Because different types of brain tissue absorb and release different amounts of energy, the tissues appear distinct on the resulting magnetic resonance image (for further detail, see the article by Doria, pp. 261–265).

This procedure has proven valuable in the study of prenatal alcohol exposure because it allows a clear and detailed picture of the brain without any dangerous or painful procedures. Upon clinical inspection, one can see obvious anomalies, such as erroneous neuron migration or an absence or thinning of portions of the corpus callosum. With further computer analysis, the brain can be visually dissected and the areas or volumes of various brain structures quantified. By comparing the sizes of various brain structures in alcohol-exposed children versus nonexposed control children, one can determine which parts of the brain are more affected than others by prenatal alcohol exposure.

### Case Studies

To date, only a few MRI studies of children with PEA or FAS have been reported in the literature; these include both case studies and group studies. Case studies typically review the findings from one or several cases and describe the children’s demographic and physical characteristics in detail. Usually these studies provide descriptions of the clinical findings (e.g., agenesis of the corpus callosum) in each case. In contrast, group studies use more detailed computer analyses to evaluate the MRI’s. In these studies, researchers compare the combined data from a group of alcohol-exposed children with data from a group of nonexposed children, trying to determine statistically whether any significant differences exist between the two samples.

At this time, six case studies in children with FAS or PEA have included results from magnetic resonance images. These reports primarily have included clinical analyses of the MRI data, such as descriptions of brain damage consistent with the spectrum of holoprosencephaly, and measurements of reductions in the size of specific brain areas. The case studies suggest that the brain areas particularly affected by alcohol are the cerebral cortex, cerebellum, basal ganglia, hippocampus, and corpus callosum. These areas comprise much of the total brain, but as discussed below, selected areas appear more affected than others by PEA.

#### Case Reports of Severely Affected Children

The first study using MRI to evaluate a child with FAS was included in a report on children with “malformative” syndromes and mental retardation (Gabrielli et al. 1990). Only one child with FAS was included, and only minimal information was provided. The child’s brain was described as displaying relatively diffuse brain damage. Shortly after this initial report, three children of alcoholic women were examined (Ronen and Andrews 1991). The brains of all three children displayed brain damage along the spectrum of holoprosencephaly. Each child was severely affected, and one died early in life. An additional child with FAS was included in a case report describing two children with corpus callosum abnormalities (Schaefer et al. 1991). This child also was severely affected and died at 6.5 months of age. An MRI prior to her death and a subsequent autopsy (Coulter et al. 1993) revealed severe and widespread brain damage, including abnormalities of the corpus callosum, basal ganglia, diencephalon, hippocampus, and cerebellum. Again, these case reports are valuable, but like the autopsy reports, they probably represent the more severe end of the spectrum of alcohol’s effects. In spite of this bias, a common thread may run through these reports: a pattern of damage that conforms to the spectrum of holoprosencephaly. Whether this is a true pattern or simply an artifact of the global and diffuse brain damage resulting from FAS remains to be determined, possibly by comparing FAS children with groups of children who have similar levels of brain damage unrelated to alcohol exposure (e.g., children with Down syndrome).

#### Reports on Higher Functioning Children

In addition to the clinical case reports of severely affected children with FAS, a few case reports of higher functioning children have been documented. The MRI’s of two adolescent children with FAS were described in a case report published in 1992 (Mattson et al. 1992). Although these children were definitely affected by their exposure to alcohol during gestation, they had survived the perinatal period and were functioning at the level of moderate mental retardation (i.e., the two children had IQ’s of 51 and less than 41, respectively).^3^ The MRI’s of these two children were evaluated using a detailed analysis in which the volumes of specific brain structures were compared with those of normal control subjects (i.e., a volumetric analysis was conducted). Both children had abnormalities of the corpus callosum; in one, the structure was missing (i.e., complete agenesis), and in the other, the callosal body was thin in certain areas. The MRI’s also revealed that the normal fluid-filled spaces in the brain (i.e., the ventricles) were enlarged, and volumes of the total brain, cerebellum, basal ganglia, and diencephalon were reduced. Furthermore, the children with FAS were compared with a small group of children with Down syndrome who resembled the alcohol-exposed children in the general level of intellectual functioning and degree of microcephaly. The brains of the FAS children showed reductions in the volumes of the basal ganglia and diencephalon when compared with the brains of the Down syndrome children. This finding suggests that prenatal alcohol exposure may particularly affect the growth of these structures. It also emphasizes that mental retardation and microcephaly (conditions that are present in both FAS and Down syndrome) are not sufficient to cause the pattern of brain damage seen in FAS.

Subsequent to this case report, two additional children were described in the literature. These children were exposed to high levels of alcohol during prenatal development but did not meet the traditional criteria for a diagnosis of FAS. The MRI’s of these PEA children indicated that the basal ganglia were reduced in volume compared with normal control children, even when brain size was taken into account. The diencephalon also was smaller, but not when viewed as a proportion of the total brain size.

## Group Studies Evaluating Brain Structure in Children With FAS and PEA

In addition to these case reports, a few studies have evaluated MRI results in groups of children with FAS or PEA. These studies have focused, for the most part, on detailed volumetric analyses of the brain. Thus far, the total brain, cerebellum, cerebellar vermis (which is part of the cerebellum), ventricles, basal ganglia, diencephalon, and corpus callosum have been measured. The findings from these reports are summarized below (Mattson et al. 1992, 1994; Riley et al. 1995; Sowell et al. 1996).

### Effects on Overall Brain Size

Children with FAS typically are microcephalic. This characteristic has been known since the initial case reports of FAS and is often mentioned as one of the diagnostic criteria. Recent MRI studies have confirmed this finding, revealing that total cranial capacity is reduced in children with FAS and PEA.

### Effects on Cerebellar Size

Results of MRI studies have revealed that in addition to reductions in the total volume of the brain, the size of the cerebellum also is reduced. Of 10 alcohol-exposed children who have been studied thus far, 8 have shown reductions in this brain region. Another study evaluated three regions of the cerebellar vermis in nine children with FAS or PEA (Sowell et al. 1996). In the children with either FAS or PEA, the vermal region located the farthest forward (i.e., the anterior vermis), but not the other regions, was reduced compared with that of normal control children. Studies of rats exposed to alcohol during the neonatal period have shown similar results. Specifically, the anterior vermis was reduced in the alcohol-exposed rats as a result of a decrease in the number of large, specialized cerebellar nerve cells called Purkinje’s cells (Goodlett et al. 1990; West et al. 1990) in this part of the brain. Although the number of cells has not been evaluated in humans, it may be that a similar loss of cells accounts for the reductions in the anterior vermis seen in children with FAS or PEA.

### Effects on Ventricular Size

In children with FAS or PEA, the ventricles are enlarged. This increase in size may be related to the reduction in brain size mentioned earlier. In other words, decreases in brain tissue may go hand-in-hand with increases in fluid-filled spaces. Whether the increase in ventricular volume noted in children with FAS or PEA is caused by the birth of fewer brain cells or an increase in the rate of cell death is not known. However, the volume of fluid that surrounds the brain within the cranial vault also has been found to be increased in a few children with FAS. Because the size of the vault of the cranium is determined, at least in part, by the growth of the brain, additional fluid surrounding the brain suggests increased cell death—that is, the brain grew, determined the size of the cranial vault, then shrank in volume, possibly as a result of cell death. Alternatively, changes in both cell death and cell birth may contribute to these reductions in brain size in FAS children.

### Effects on the Basal Ganglia and Diencephalon

**Figure 2:**
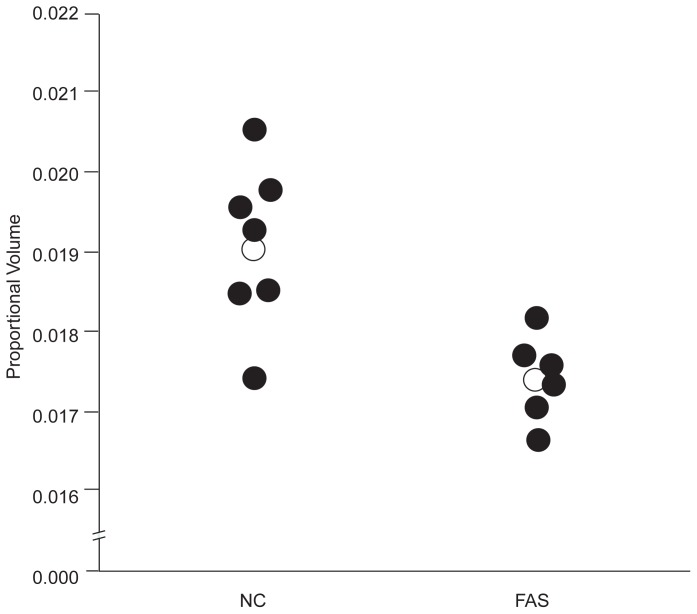
Graph depicting the proportional volumes of the basal ganglia of six children with fetal alcohol syndrome (FAS) and seven normal control children (NC). Each circle represents one child, and the open circles represent the group average. The children with FAS show a reduction in ganglia volume.

The basal ganglia and diencephalon are groups of structures that lie deep within the brain and, as mentioned earlier, are associated with both cognitive and motor abilities. Initially, children with FAS appeared to show reductions in both these brain regions. In the first two FAS children evaluated by Mattson and colleagues (1992), both of these brain regions were reduced when compared with those of normal children. Later studies revealed that when total brain size was controlled to account for the FAS children’s microcephaly, the diencephalon was not disproportionately smaller in the FAS children than in the control children (Mattson et al. 1994; Mattson and Riley in press). This finding indicates that although the diencephalon is reduced in FAS subjects when compared with control subjects, it is probably a result of the reduction in brain size caused by the alcohol exposure. Therefore, the diencephalon probably is not more sensitive to prenatal alcohol exposure than the brain as a whole. In contrast, even when total brain size is accounted for, the volume of the basal ganglia in FAS subjects is disproportionately reduced when compared with that of the control subjects. That is, even when the basal ganglia are viewed as a proportion of total brain size or when equally microcephalic control subjects are used (e.g., children with Down syndrome), this group of structures continues to be smaller than normal. This result suggests that the basal ganglia may be especially sensitive to the effects of prenatal alcohol exposure (see figure 2).

Further studies have revealed that the total reduction in the basal ganglia may be primarily attributable to reductions in the caudate nucleus. Specifically, when the basal ganglia are separated into subsections (i.e., the caudate and lenticular nuclei), the reduction in the caudate nucleus is significant, whereas that of the lenticular nuclei is not. The caudate nucleus is traditionally associated with cognitive functioning, whereas the putamen (part of the lenticular region) may be more important for motor functioning. This result may help explain some deficits suffered by children with FAS or PEA, but further study and replication are required before any conclusions can be reached.

### Effects on the Corpus Callosum

**Figure 3:**
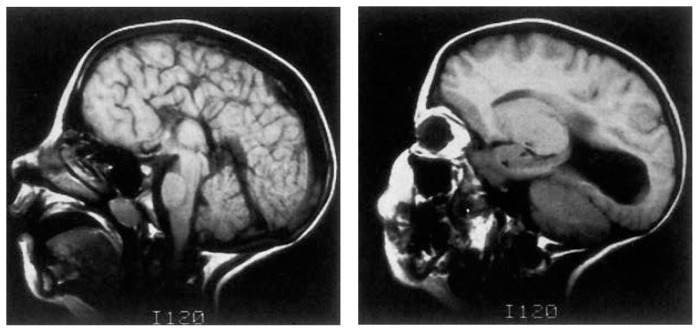
Two magnetic resonance images from a child with fetal alcohol syndrome demonstrating agenesis of the corpus callosum (left) and colpocephaly (right), a feature often associated with callosal agenesis.

The corpus callosum allows information to travel between the brain’s two hemispheres and permits the two halves to work together. Some of the earliest reports of brain damage in children with FAS described complete agenesis of the corpus callosum. This brain abnormality occurs infrequently in the normal population,^4^ at a rate of about 1–3 per 1,000 (0.1–0.3 percent) (NIAAA 1990). In the developmentally disabled population, the incidence increases to about 2.3 percent (Jeret et al. 1986). In FAS and PEA, however, the incidence may be even higher. Out of 44 children in Mattson and colleagues’ sample (e.g., Riley et al. 1995), the researchers are aware of 3 children (6.8 percent) who have callosal agenesis (see figure 3).

In addition to reports of corpus callosum agenesis, information also is available on the size of this structure in children with FAS or PEA (Riley et al. 1995). Ten children with FAS or PEA, excluding the children with agenesis, were evaluated using the area of a photographic slice of the corpus callosum.^5^ In these 10 children, the area of the slice was smaller than that of normal control children. Further analyses were conducted by dividing the area of the corpus callosum into five parts. When brain size was accounted for by measuring the callosal area as a proportion of brain area, three of five callosal regions were reduced in size compared with those of control subjects. The most anterior region and the two posterior regions of the corpus callosum were significantly different (i.e., statistically) from that of the control subjects. Notably, similar findings have been reported in children with attention deficit hyperactivity disorder (Hynd et al. 1991).^6^ Given the common occurrence of attention deficits in children with FAS, this overlap may lead to further understanding of the etiology of attentional deficits and callosal abnormalities in both alcohol-exposed and nonexposed children.

## Significance of Findings

As the findings describe above, alcohol can have devastating consequences on the developing fetus. MRI and other research conducted thus far suggest that several parts of the brain are reduced in size following prenatal alcohol exposure. But not all the regions evaluated thus far have shown specific size reductions; although the basal ganglia and corpus callosum appear to be disproportionately smaller in alcohol-exposed children, the diencephalic structures diminish in proportion to the reduction in total brain size. In another component of this pattern, the anterior vermis of the cerebellum appears to be disproportionately reduced in size relative to other vermal areas.

The full significance of the findings from MRI-based research remains unclear. Although we know that certain brain areas—such as the basal ganglia, cerebellum, and corpus callosum—appear to be especially sensitive to prenatal alcohol exposure, we do not yet know what functional consequences these size reductions may have. Similarly, although other brain areas (e.g., the diencephalon) are reduced in volume only as much as the brain is reduced in size, these volume reductions also may have functional consequences. Although not emphasized here, the FAS and PEA children that Mattson and colleagues have evaluated and described certainly have cognitive deficits. For the most part, their cognitive ability falls below the normal range. Children with FAS typically are mildly mentally retarded, with an average IQ of about 70. Ability levels range considerably, however, and IQ’s have been reported between 20 and 120 (the average IQ for the general population is 100). In addition, specific cognitive deficits have been reported in several domains, including language, attention, verbal learning, and memory.

The relationship between the reported cognitive deficits and brain abnormalities is not yet known, although some degree of correlation can be assumed. Mental retardation in itself, however, does not indicate what pattern of brain damage exists. For example, when FAS children were compared with Down syndrome children, both groups had similar levels of mental retardation and microcephaly. However, a different pattern of brain abnormality was observed in each of the two groups. The children with FAS had reductions in the basal ganglia, but the children with Down syndrome showed relatively minimal damage in this brain region, even given their microcephaly. These results suggest that mental retardation and microcephaly cannot predict reductions in the basal ganglia and further emphasize the sensitivity of the basal ganglia to prenatal alcohol exposure. The differences in brain development may be related to differences in cognitive strengths and weaknesses exhibited by the two groups.

## Future Directions

As scientists learn more about the cognitive deficits and brain abnormalities in FAS and PEA, they will need to link these two areas of research. Although relating brain structure and function is difficult both in theory and practice, this connection will reveal the most about the brain and how it is affected by alcohol. The brain differences now being discovered between alcohol-exposed and normal control children are exciting and interesting. However, until researchers know what these differences mean for individual children with FAS, their capacity to provide assistance is limited. An understanding of which brain areas are affected may enable researchers in the field to develop hypotheses about brain chemistry and suggest pharmacological treatments for children with FAS. MRI research may help solve questions that arise even from children with mild cognitive deficits resulting from minimal alcohol exposure during pregnancy. The field of brain chemistry research also requires further study. Imaging techniques, such as functional MRI or positron emission tomography—which evaluate brain activity as people perform tasks (for further details, see the article by Doria, pp. 261–265)—may soon make it possible to determine what the neurochemical parameters are in children with FAS. Neuropsychological assessment is another valuable tool in evaluating alcohol’s teratogenic effects. By testing different skills, such as learning and memory, neuropsychology allows both practical and theoretical understanding of the real abilities and disabilities of children with FAS and PEA. Such knowledge may lead to the design of remediation strategies useful to teachers and other caregivers. In addition, MRI may ultimately become useful as a diagnostic tool among prenatally exposed children in whom the facial features signaling FAS are absent.

## Summary

FAS is a devastating disorder that affects children prenatally exposed to high levels of alcohol. This alcohol exposure causes abnormalities in a child’s physical as well as psychological well-being. Specifically, the developing brain appears to be especially sensitive to prenatal alcohol exposure, the effects of which are permanent. Regions that may show increased sensitivity to this exposure include the cerebellum, corpus callosum, and basal ganglia. The functional consequences of this pattern of brain damage have yet to be uncovered, although a fair amount is known about the cognitive abilities of children with FAS. Future research will continue to focus on uncovering the brain abnormalities resulting from prenatal alcohol exposure and determining the relationships between these abnormalities and everyday functioning. By understanding these relationships, researchers hope to further knowledge of how alcohol’s effects can be prevented and treated.

## Figures and Tables

**Figure 1:**
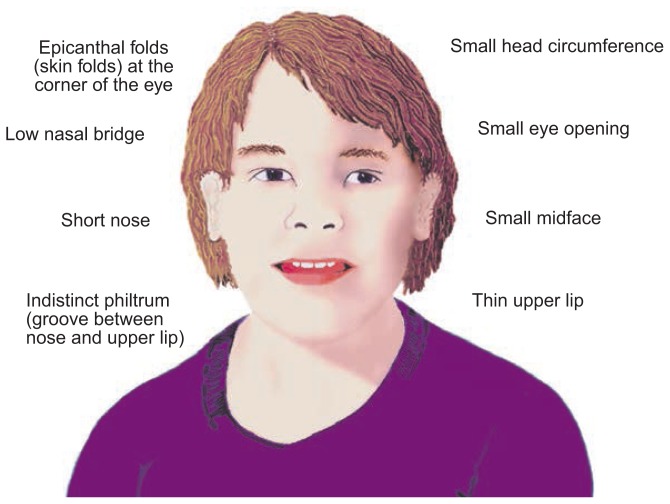
Illustration of the craniofacial features associated with fetal alcohol syndrome.

## Footnotes

1 The extent of alcohol exposure to a fetus during pregnancy can vary greatly. It is not known, however, how much alcohol exposure is required before effects on the fetus become noticeable. Because this amount probably varies from one mother-fetus pair to another, the National Institute on Alcohol Abuse and Alcoholism (NIAAA) advocates that all women abstain from alcohol consumption during pregnancy.

2 For definitions of this and other brain areas, see diagrams and glossary, pp. 293–295.

3 The average IQ in the population is 100. An IQ of less than 70 is usually considered to reflect mental retardation, and IQ scores of 70 to 80 are considered to be in the borderline range.

4 The term “normal population” refers in general to people with no cognitive abnormalities or people who have not been referred to a professional for evaluation resulting from any such anomaly.

5 For this finding, the corpus callosum was measured in the midsagittal section, a slice along the middle of the structure looking from the side of the head.

6 The term “attention deficit hyperactivity disorder” is defined by the American Psychiatric Association’s *Diagnostic and Statistical Manual of Mental Disorders, Fourth Edition*, as a “persistent pattern of unattention and/or hyperactivity.”
